# The Relationship Between Temperament, Screen Exposure, and Psychological Adjustment in Preschool Children

**DOI:** 10.3390/children13060721

**Published:** 2026-05-22

**Authors:** Barbara Jelić, Dario Vučenović, Jelena Flego

**Affiliations:** 1 Kindergarten Mali Svijet, Borovik 5, Lucko, 10000 Zagreb, Croatia; barbarajelic317@gmail.com; 2 Department of Psychology, Faculty of Croatian Studies, University of Zagreb, Borongajska Cesta 83d, 10000 Zagreb, Croatia

**Keywords:** temperament, screen exposure, psychological adjustment, preschoolers

## Abstract

**Highlights:**

**What are the main findings?**

- Impulsivity showed the strongest associations with all indicators of psychological adjustment.
- Weekend screen time moderated the relationships between certain temperament dimensions and behavioral outcomes.

**What are the implications of the main findings?**

- Screen exposure may shape how temperament is expressed in early childhood.
- Interventions should consider both child temperament and patterns of screen use.

**Abstract:**

Objectives: The aim of this study was to analyze current trends in screen exposure and to provide a deeper understanding of the relationships between temperament, screen exposure, and psychological adjustment in preschoolers. Methods: The study was conducted in kindergartens and one health center in the city of Zagreb, using a convenience sample of 115 mothers who assessed their preschool children’s screen exposure, temperament, and psychological adjustment. Results: Descriptive data analysis indicated that children’s screen time generally fell within the American Academy of Child and Adolescent Psychiatry’s recommended guidelines. Correlation analysis indicated that externalizing problems were significantly positively correlated with impulsivity, activity, emotionality, and weekend screen time. Conversely, prosocial behavior was negatively correlated with impulsivity and weekend screen exposure. Moderation analyses revealed that weekend screen time significantly altered the associations between temperament and externalizing problems. Specifically, longer weekend screen exposure weakened the relationships between Impulsivity and externalizing problem and between Activity and externalizing problems, suggesting that screen time may buffer the impact of high-risk temperament profiles on behavioral difficulties. Weekend screen time did not moderate the relationship between Emotionality and externalizing problems. Similarly, longer screen exposure weakened the negative association between Impulsivity and prosocial behavior, indicating that screen time may reduce the extent to which impulsive temperament undermines prosocial functioning in preschool children. Conclusions: These findings provide deeper insight into the role of temperament and screen time exposure in predicting both maladaptive and prosocial behaviors among preschool-aged children.

## 1. Introduction

Early childhood is a key time for socio-emotional development, shaped by both biology and environment. One widely studied trait is temperament, referring to stable differences in emotional reactions, energy, attention, and regulation that emerge early in life [1,2]. This study used Buss and Plomin’s EAS model [2], which has three main areas: Emotionality, Activity, and Sociability. Emotionality refers to how easily a child becomes upset. Activity is the child’s overall energy and pace in daily life. Sociability is the tendency to be around others [3]. These traits are usually stable over time and can predict later personality and behavior [4]. The model later added Impulsivity, which refers to how well a child can control emotional or impulsive responses [5].

Children today are surrounded by digital devices and screens every day. Screen exposure encompasses the time spent on TVs, smartphones, tablets, computers, or game consoles, and it starts early. For example, European studies show that preschoolers watch TV for 20 min to 4 h a day, with more screen time on weekends [6]. In Croatia, children spend an average of about 2 h and 42 min per day on weekdays and over 3 h per day on weekends in front of various screens [7]. Many young children also use YouTube, social media, and tablet games. These numbers show that digital media is a big part of children’s daily lives. These effects can be described by the displacement hypothesis, which holds that time spent on screen media necessarily reduces time available for other developmentally beneficial activities, such as peer play, reading, and physical activity [8]. Digital environments are increasingly influential in early childhood, prompting researchers to examine how screen exposure may relate to children’s developmental outcomes. Psychological adjustment, commonly defined as the ability to adapt successfully to various demands while maintaining age-appropriate functioning, is an important indicator of children’s well-being [9]. In early childhood, psychological adjustment can usually be assessed by examining prosocial behavior and internalizing and externalizing problems, as seen in past research [10]. In line with this literature, in the present study, we use the term psychological adjustment to refer to this constellation of outcomes, operationalized through mother-reported prosocial behavior and internalizing and externalizing problems.

Previous research suggests temperament may play a key role in behavioral adjustment. For example, higher negative emotionality is associated with an increased chance of externalizing problems, particularly when accompanied by lower behavioral regulation [11]. Similarly, Oland and Shaw [12] state that high social withdrawal, anxiety, and inhibition, along with high impulsivity, hyperactivity, and emotional rejection, can make it more difficult to achieve important social developmental milestones, which in turn may later increase the risk of internalizing and externalizing problems in adolescence. From a developmental perspective, individual differences in temperament are likely to shape how children engage with screen media. A negative correlation has been found between effortful control and screen exposure in children aged 3 to 5 [13], suggesting that greater screen exposure is associated with lower effortful control. Fitzpatrick et al. [14] found that higher screen exposure at three and a half years predicted lower effortful control at four and a half years, but lower effortful control at three and a half years did not predict later higher screen exposure. Moreover, children who showed higher results on temperament dimensions indicating difficult temperament were allowed to start watching television at an earlier age [15]. Emotional reactivity has also been found to be positively correlated with screen time [16]. The mentioned result could indicate that allowing screen access could represent a possible coping mechanism for parents of children exhibiting more difficult temperament [16]. Finally, Tomopoulos et al. [17] reported that viewing non-educational (but age-appropriate) children’s programs was positively associated with externalizing problems in three-year-olds, suggesting that this type of screen content may contribute to such difficulties.

Other studies indicate that temperament and screen exposure independently predict prosocial behavior in children. Parker-Cohen and Bell [18] found that children exhibiting higher levels of activity and approach, along with lower sensitivity, demonstrate greater social responsiveness toward peers. Acar [19] identified a significant negative association between inhibitory control and conflict. A recent Turkish study reported a weak positive relationship between screen time and prosocial behavior, as well as a positive association between having a television in the bedroom and prosocial outcomes [20]. Furthermore, Gentile et al. [21] demonstrated that playing prosocial video games predicts helping behavior, cooperation, sharing, empathy, and emotional awareness among school-aged children. Growing evidence also suggests that environmental factors, such as media exposure, may affect children’s behavioral outcomes. Specifically, studies report links between long screen exposure and a higher prevalence of both internalizing and externalizing problems in preschool children [22]. Supporting these findings, a meta-analysis by Eirich et al. [23] found a small but statistically significant link between screen exposure and both problem types.

The literature on temperament, media exposure, and children’s adjustment is growing. However, few studies have examined how these factors operate within a single research framework. Understanding how stable temperamental characteristics interact with environmental influences, such as screen exposure, may provide a fuller explanation of early psychological adjustment. Accordingly, the present study aims to examine the relationship between temperament, screen exposure, and psychological adjustment in preschool children. Based on previous research, several directional hypotheses were formulated. Higher Emotionality and Impulsivity were expected to be positively associated with externalizing and internalizing problems. In contrast, Sociability was expected to be positively associated with prosocial behavior and negatively associated with internalizing problems. Greater screen exposure was expected to be positively associated with externalizing and internalizing problems. However, for relationships where empirical evidence remains mixed or limited—specifically regarding Activity, the link between screen exposure and prosocial behavior, and the moderating role of screen exposure—non-directional associations were hypothesized. It was expected that these variables would be significantly associated with psychological adjustment outcomes, although the direction of these relationships would be explored empirically.

## 2. Materials and Methods

### 2.1. Participants

A total of 115 mothers participated in the study. Nine participants were excluded from the analyses based on a screening item related to the child’s age. Only mothers of children aged 6 years or older were included. This criterion matches the Croatian school enrollment regulation, which allows children who turn 6 by 1 April to enter first grade in the following school year [24]. Mothers’ ages ranged from 26 to 60 years (M = 40.50, SD = 5.34). Educational level in the sample can be seen in Table 1.

Most participants were currently employed (N = 92). Nine mothers reported being unemployed or on maternity/parental leave. Two mothers had caregiver status. One participant was on childcare leave, and another on adoption leave. Work experience ranged from 3 to 29 years (M = 14.71, SD = 5.86). Family Income level is shown in Table 2.

Most mothers (91.6%) lived in cities with more than 100,000 inhabitants. Another 7.5% lived in towns with 10,000–100,000 inhabitants. Only 0.9% lived in settlements with fewer than 2000 inhabitants. About 89% reported living in two-parent households, while 11% reported living in single-parent households. One respondent chose “other” and specified “parent and child,” which was categorized as a single-parent household. The number of children per family ranged from one to four (M = 2.05, SD = 0.76). Household size ranged from one to seven members (M = 3.91, SD = 0.94). In 47.7% of cases, mothers reported on a male child, and in 52.3% on a female child. Children’s ages ranged from 6 to 8 years (M = 6.41, SD = 0.46). A total of 81.3% of mothers reported that their child had not been granted a delayed start to primary school, while 19.7% reported that their child had been granted a delayed start. Participants were recruited from among mothers of children scheduled to start primary school in the following year, in accordance with the Croatian education system. Therefore, despite their higher chronological age, all participants were considered part of the preschool cohort based on their educational status.

### 2.2. Instruments

#### 2.2.1. Socio-Demographic Questionnaire for Mothers and Children

A socio-demographic questionnaire was developed for the purposes of this study. It included items assessing characteristics of both mothers and children. The first section covered maternal characteristics (age, education level, employment status, household income, and number of children), while the second section focused on child characteristics (age, gender, and potential school-start delay). The questionnaire included both open-ended items (e.g., age) and closed-ended items in multiple-choice or dichotomous (yes/no) formats.

#### 2.2.2. Screen Exposure Questionnaire

A questionnaire assessing children’s screen exposure was developed for this study. It captured the broad construct of screen exposure through several subscales based on previous research [7,25,26]. The first subscale included seven items assessing the presence of devices in the household (television, smartphone, tablet, laptop, desktop computer, gaming console, and virtual reality headset), with yes/no responses. The second part assessed frequency of device use during the past week using a 5-point Likert scale (1 = never to 5 = very often). An additional dichotomous item assessed whether the child owns a personal smartphone. The final two open-ended items assessed total screen time during the past week and weekend separately. Prior studies have commonly relied on a limited number of items to assess children’s screen time [27], whereas previous research has specifically differentiated between weekday and weekend exposure [7]. Accordingly, screen time was assessed separately for weekdays and weekends in the present study.

#### 2.2.3. EASI Child Temperament Questionnaire

The Croatian version of the EASI questionnaire [28], based on the original instrument by Buss and Plomin ([2], as cited in Rowe and Plomin [29]), was used to assess four temperament dimensions: Emotionality, Activity, Sociability, and Impulsivity. The instrument comprises 20 items and is divided into four subscales. Responses are provided on a 5-point Likert scale (1—“Not at all”; 2—“Mostly not”; 3—“Neither yes nor no”; 4—“Mostly yes”; 5—“Always true”), through which parents assess the frequency of specific types of behavior in their children. However, 6 items are reverse-coded, which requires recoding during data processing. Scores range from 5 to 25 points for each subscale and are calculated using a linear combination of the ratings on the respective subscale. In this study, Cronbach’s alpha coefficients were 0.74 for Emotionality, 0.69 for Activity, 0.54 for Sociability, and 0.65 for Impulsivity. Overall questionnaire reliability was acceptable (α = 0.76).

#### 2.2.4. Strengths and Difficulties Questionnaire (SDQ)

The Strengths and Difficulties Questionnaire (SDQ) [30] was used to measure children’s psychological adjustment through the assessment of prosocial behavior, internalizing problems, and externalizing problems. The Croatian parent-report version of the questionnaire for children aged 4–17 years, which was translated by Helena Hamilton and Natasa Momcilovic [31], was used in this study. The questionnaire consists of 25 items measuring five dimensions: hyperactivity, conduct problems, emotional symptoms, peer problems, and prosocial behavior. Responses are rated on a 3-point scale (0–2). Subscale scores range from 0 to 10, and a total difficulties score (0–40) can be calculated. Due to reliability concerns in this sample, broader theoretically informed composites were created following SDQ scoring guidelines: internalizing problems (sum of the emotional symptoms and peer problems subscales, α = 0.63) and externalizing problems (sum of the conduct problems and hyperactivity subscales, α = 0.67), and prosocial behavior (α = 0.72). The use of these combined scales is recommended over individual subscales in low-risk community samples [31].

### 2.3. Procedure

The study was conducted in five public kindergartens in Zagreb and one primary healthcare center where school readiness assessments were being performed. The sample can therefore be considered a convenience sample. Data collection took place in June of 2024 and lasted approximately 3.5 weeks. Questionnaires were administered in group settings using paper-and-pencil methods and took 5–20 min to complete. Participants were informed in advance via official kindergarten communication channels (WhatsApp groups and mailing lists). Upon arrival, mothers joined the data collection process and provided written informed consent before participation. Anonymity was ensured. Data collection was conducted by psychology students in collaboration with kindergarten psychologists. Although conditions were largely standardized, high temperatures (occasionally exceeding 31 °C), limited parking availability, parental time constraints, and reduced attendance due to approaching summer vacations represented potential sources of unsystematic variance. Completed questionnaires were manually entered into the IBM SPSS Statistics program (Version 31.0.2.0; IBM Corp., Armonk, NY, USA). Hard copies were securely stored by the research team, while digital data were saved on a USB device. During the preparation of this manuscript, the authors used ChatGPT (OpenAI), Google Gemini (Google), Perplexity (Perplexity AI) and Grammarly (Grammarly Inc., San Francisco, CA, USA) for translation, language editing, clarity improvement, and refinement of academic expression. The authors have reviewed and edited the output and take full responsibility for the content of this publication.

### 2.4. Statistical Analyses

Data were analyzed using the IBM SPSS Statistics program. Descriptive statistics, Pearson correlation, and hierarchical multiple regression analyses were carried out to examine the study variables and their interrelations. First, we computed descriptive indices for screen exposure, temperament dimensions, externalizing and internalizing problems, and prosocial behavior. Next, bivariate correlations were calculated to examine associations among these constructs.

To address the research questions regarding moderation, we ran four separate hierarchical regression models: (1) weekend screen time moderating the association between Impulsivity and externalizing problems, (2) weekend screen time moderating the association between Activity and externalizing problems, (3) weekend screen time moderating the association between Emotionality and externalizing problems, and (4) weekend screen time moderating the association between Impulsivity and prosocial behavior. Before the analysis, all continuous predictors were mean-centered to reduce multicollinearity. In each model, the main effects of temperament and weekend screen time (centered) were entered in Step 1, followed by the interaction term (temperament × weekend screen time) in Step 2. Variance inflation factors (VIFs) were examined for all predictors and indicated no concerns regarding multicollinearity.

To address missing data, descriptive statistics and bivariate correlations were calculated using pairwise deletion to maximize the use of available data for each variable or variable pair. Moderation analyses were executed using a complete-case (listwise deletion) approach, in which only participants with data available for all variables included in a given model were retained. As a result, the sample size varied across analyses. Missing data were mainly due to incomplete questionnaire responses.

## 3. Results

### 3.1. Descriptive Analysis of Screen Exposure

**Figure 1:**
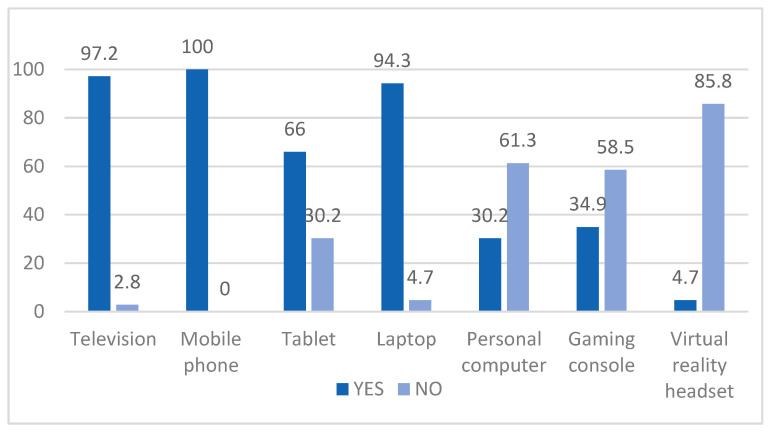
Device ownership in the household (%) (N = 106).

The analysis of descriptive data on screen exposure began with an overview of the frequencies of “YES” and “NO” responses regarding household device ownership. As shown in Figure 1, almost every household owned a television (97.2%) and a mobile phone (100%). Furthermore, analysis of the dichotomous item on possession of a personal mobile device among children showed that 14.3% of the preschool sample owned a mobile device.

The results presented in Table 3 indicate frequent exposure to television (C = 4, q = 2), rare use of mobile phones (C = 2, q = 2), and a lack of exposure to other devices, including tablets, laptops, personal computers, gaming consoles, and virtual reality headsets (C = 1, q*_T_* = 2, q*_L_*_;*PC*;*GC*;*VR*_ = 0). Additional checks of the distribution of responses confirmed the observed values, with a pronounced negative skewness in television exposure, indicating a tendency toward higher values. In other words, parents most often reported that children had frequent or very frequent exposure to television. Regarding mobile phone use among preschool children, the distribution showed positive skewness, indicating that responses tended to fall at the lower end of the scale. More precisely, half of the sample of parents responded “Not at all” or “Rarely” to the question about the frequency of mobile phone use among their children. This indicated that at least half of mothers from Zagreb reported that their child watched television five to six times during the past week, used a mobile phone once or twice, and almost never used other devices. However, it is also necessary to consider the other half of mothers, whose responses differed greatly. More precisely, the observed response ranges suggest that children in the sample used all devices. At the same time, a detailed inspection of frequencies confirmed that at least one mother on each of the items (except for laptops, personal computers, and virtual reality headsets) reported that her child used a specific device “Very often”, that is, 6–7 times per week.

As seen in Table 4, descriptive analyses were conducted on the dimensions of temperament (Emotionality, Activity, Sociability, and Impulsivity), time-based screen exposure, and the constructs of psychological adjustment (internalizing problems, externalizing problems, and prosocial behavior). Based on the S-W normality test results in Table 4, the distributions of Emotionality, Activity, and Impulsivity were found to be normal, indicating that most temperament dimensions were normally distributed in the sample of Zagreb preschool children. The Sociability dimension is the only one that showed a significant deviation from normality (S-W = 0.95, *p* < 0.01) with negative skewness (S = −0.85), indicating a tendency toward higher scores and suggesting that most children displayed high sociability. Emotionality, Activity, and Impulsivity showed moderate-to-high mean scores (M = 12.07 − 15.56), indicating a medium-to-high expression of these dimensions among Zagreb preschool children.

The variables internalizing problems, externalizing problems, prosocial behavior, weekday screen time, and weekend screen time show significant results on the S-W test, suggesting deviations from normality. The reason may lie in the S-W test’s pronounced sensitivity, which gives it a clear advantage over other tests for assessing normality [32]. Given the significant deviations of the distributions from normality, indices of skewness and kurtosis, as well as histograms of the aforementioned constructs, were also analyzed, which is considered a justified way to assess the fulfillment of the conditions for conducting parametric statistical analyses according to Petz [33]. Visual inspection of the histograms indicated that the internalizing problems, externalizing problems, and total difficulties are positively skewed, suggesting a greater tendency toward lower scores and, at the same time, a reduced expression of these problems among Zagreb preschool children. Furthermore, the distribution of responses for the prosocial behavior variable fell toward higher values, suggesting a high proportion of preschool children displaying prosocial behavior. This is considered justified because in studies on low-risk children, that is, children within a non-clinical population, parents assess lower levels of internalizing and externalizing problems and higher levels of prosocial behavior [34,35]. The distributions of responses for exposure during the week and on the weekend suggest that most children are exposed to screens for several hours, so both distributions are positively skewed. Analysis of skewness and kurtosis indices indicates slight deviations from the distributions. More precisely, the distributions are slightly positively skewed, indicating a tendency for the results to be medium to low for both screen exposure variables (M = 5.34; M = 2.64). However, such distributions are expected given the guidelines of the American Academy of Child and Adolescent Psychiatry (AACAP) [36]. Furthermore, the median duration of exposure was calculated, yielding C = 5 (q = 5) for the week and C = 2 (q = 3) for the weekend. Given that this is a ratio scale and that there are no drastic differences between the median and means on both measures, we consider it appropriate to use the obtained means in further parametric analyses.

To summarize, despite significant Shapiro–Wilk test results, parametric analyses (Pearson correlations and hierarchical regression) were deemed appropriate for several reasons. Firstly, the sample size (N = 103–106 for correlations; N = 103–104 for moderation analyses) substantially exceeded the robustness threshold (N > 30) beyond which parametric tests remain robust to violations of normality (Elliott & Woodward, 2007; Pallant, 2007, as cited in [37]). Secondly, regression diagnostics confirmed that assumptions of linearity, homoscedasticity, and normality of residuals were met for all moderation models (see Section 3.3). Finally, distributions reflected theoretically meaningful patterns (floor effects for adjustment problems, ceiling effects for prosocial behavior) rather than measurement error [34,35]. These considerations support the use of parametric procedures to address the research questions.

### 3.2. Correlation Between Temperament, Screen Exposure, and Psychological Adjustment

In accordance with the study’s aim, analyses were conducted to examine the relationships among dimensions of temperament, time-based screen exposure, and children’s psychological adjustment.

The correlation analysis presented in Table 5 shows that externalizing problems are positively correlated with Emotionality, Activity, and Impulsivity. More precisely, externalizing problems showed a significant, weak, and positive correlation with the Emotionality dimension (r = 0.35, *p* < 0.01), indicating that higher emotionality was associated with greater externalizing problems in preschool children. Similarly, the Activity dimension showed a significant moderate positive correlation with externalizing problems (r = 0.47, *p* < 0.01), indicating that as the child’s activity increases, the number of externalizing problems also increases. However, externalizing problems were most strongly, positively, and significantly associated with the child’s Impulsivity (r = 0.67, *p* < 0.01), such that more impulsive children displayed more externalizing problems. In other words, as scores on the Impulsivity measure increased, scores on the measure of externalizing problems increased accordingly. It can also be observed that externalizing problems were positively correlated with all measured dimensions of temperament, except for the Sociability measure, which was not statistically significant. A significant correlation was also observed between externalizing problems and weekend screen exposure duration (r = 0.22, *p* < 0.05). This correlation indicates a weak positive association: as weekend screen exposure increases, the score on the externalizing problems measure also increases.

As shown in Table 5, the analysis of the relationship between internalizing problems and Emotionality indicates a significant but weak positive association (r = 0.34, *p* < 0.01), suggesting that children with a more pronounced Emotionality dimension also achieve higher scores on the internalizing problems measure. A similar relationship is observed between Impulsivity and internalizing problems, with children with higher Impulsivity displaying more internalizing problems. Namely, a significant positive and moderate association between these constructs is again observed (r = 0.39, *p* < 0.01). It was also observed that internalizing problems show a significant moderate negative correlation with the Sociability dimension (r = −0.32, *p* < 0.01), meaning that as scores on the Sociability measure increase, scores on the internalizing problems measure decrease. More precisely, more sociable children display fewer internalizing problems in the Zagreb sample. Regarding relationships with other variables, internalizing problems do not show a significant correlation with Activity, the final dimension of temperament, nor with measures of screen exposure. Given the lack of association with exposure measures, internalizing problems were excluded from further analyses.

The results from Table 5 further show that prosocial behavior is significantly, weakly, and negatively associated with Impulsivity, such that as scores on the Impulsivity measure increase, scores on the prosocial behavior measure decrease (r = −0.23, *p* < 0.05). Prosocial behavior also shows a significant weak negative correlation with screen exposure on weekends (r = −0.22, *p* < 0.05) and on weekdays (r = −0.21, *p* < 0.05). This suggests that greater screen exposure during both weekdays and weekends is associated with a decrease in the frequency of prosocial behavior among Zagreb preschool children. However, the relationship between prosocial behavior and the duration of exposure during the week was not included in further analyses, because a subsequent inspection of the scatterplot revealed a curvilinear pattern. The association between prosocial behavior and the other dimensions of temperament (Emotionality, Activity, and Sociability) was not significant.

In summary, the correlation analysis identified Impulsivity as the temperament dimension most strongly associated with all three adjustment outcomes (externalizing problems, internalizing problems, and prosocial behavior). Weekend screen exposure emerged as a significant correlate of externalizing problems and prosocial behavior, but not internalizing problems. To further explore these relationships, moderation analyses were conducted to test whether weekend screen exposure moderated the relationships between temperament dimensions and both externalizing problems and prosocial behavior.

### 3.3. Exploring the Moderating Effect of Weekend Screen Time on the Relationship Between Temperament and Psychological Adjustment

To test whether weekend screen time moderated the association between Impulsivity and externalizing problems, a hierarchical multiple regression was conducted (Table 6). Variance inflation factors ranged from 1.07 to 1.21, which is considered satisfactory. The tolerance index ranged from 0.83 to 0.94, indicating no collinearity among the predictors. Using visual analysis of the P–P plot of regression-standardized residuals and the scatterplot of standardized residuals versus predicted values, it was determined that the assumptions for conducting regression analysis were met. The full model explained 47.4% of the variance in externalizing problems (R = 0.69, R^2^ = 0.474, F (3, 100) = 29.98, *p* < 0.01). Impulsivity was a strong positive predictor of externalizing problems (b = 0.63, SE = 0.07, β = 0.67, t = 8.63, *p* < 0.01), whereas weekend screen time was not a significant independent predictor (b = 0.04, SE = 0.13, β = 0.03, t = 0.34, *p* < 0.01). The interaction between Impulsivity and weekend screen time was significant (b = −0.08, SE = 0.04, β = −0.16, 95% CI [−0.16, −0.01], t = −2.19, *p* < 0.05), accounting for an additional 2.5% of the variance in externalizing problems (ΔR^2^ = 0.025, ΔF (1, 100) = 4.78, *p* < 0.05) with a small effect size (f^2^ = 0.05). More precisely, these data suggested that screen exposure significantly moderated the relationship between Impulsivity and externalizing problems, such that the association between the two is somewhat weaker at higher levels of weekend screen time.

To examine whether weekend screen time moderated the association between Activity and externalizing problems, a hierarchical multiple regression was conducted (Table 7). As shown in Table 7, the variance inflation factor (VIF) indices ranged from 1.01 to 1.02, indicating very low multicollinearity among the independent variables. Furthermore, high tolerance coefficients indicated low multicollinearity, allowing for further interpretation of the results. The full model accounted for 35.5% of the variance in externalizing problems (R = 0.60, R^2^ = 0.36, F (3, 99) = 18.20, *p* < 0.01). Both Activity (b = 0.38, SE = 0.07, β = 0.46, t = 5.59, *p* < 0.01) and weekend screen time (b = 0.36, SE = 0.13, β = 0.23, t = 2.80, *p* < 0.01) were significant individual positive predictors of externalizing problems. Importantly, the interaction between Activity and weekend screen time was significant (b = −0.10, SE = 0.03, β = −0.28, 95% CI [−0.16, −0.04], t = −3.39, *p* < 0.01), explaining an additional 7.5% of the variance in externalizing problems (ΔR^2^ = 0.075, ΔF (1, 99) = 11.47, *p* < 0.01) with a small-to-medium effect size (f^2^ = 0.12).

The final tested model for predicting externalizing problems involved a moderation analysis of the relationship between Emotionality and externalizing problems with respect to weekend screen exposure. Despite the significant correlations in Table 5, no significant interaction was found between screen exposure duration and Emotionality in predicting externalizing problems in Zagreb preschool children (b = −0.04, *p* > 0.05).

Table 8 presents the moderation analysis of the relationship between Impulsivity and prosocial behavior, with respect to the duration of screen exposure on weekends. The tolerance index ranged from 0.82 to 0.94, while the variance inflation factor ranged from 1.07 to 1.21. Inspection of the histogram of the criterion variable revealed minor deviations from normality, while the P–P plot of regression-standardized residuals indicated linearity. Finally, the dispersion of standardized residuals was examined, which indicates the presence of outliers. Despite these minor deviations, the model can be considered to have met the assumptions. Hierarchical multiple regression revealed a significant interaction between Impulsivity and weekend screen time in predicting prosocial behavior (b = −0.081, SE = 0.026, β = −0.298, 95% CI [−0.13, −0.03], t = −3.133, *p* = 0.002). The full model explained 15.8% of the variance, and the interaction term itself accounted for an additional 8.3% of the variance (ΔR^2^ = 0.083, F change = 9.82, *p* = 0.002) after the first step, indicating a small-to-medium effect (f^2^ = 0.10). The main effects of Impulsivity (*p* = 0.056) and weekend screen time (*p* = 0.465) were not statistically significant, suggesting that the relationship between Impulsivity and prosocial behavior depended on the level of screen exposure rather than operating independently.

To sum up, weekend screen exposure consistently moderated the relationships between temperament dimensions and psychological adjustment. Across all significant models, longer screen exposure weakened the associations between high-risk temperament traits (Impulsivity, Activity) and behavioral outcomes. Specifically, screen time buffered the impact of impulsive and active temperament on externalizing problems and reduced the negative effect of impulsivity on prosocial behavior. Emotionality did not show a significant interaction with screen time in predicting externalizing problems. These findings suggest that, in this sample, longer weekend screen exposure was associated with weaker links between high-risk temperament traits and behavioral outcomes, but these patterns should be interpreted cautiously due to measurement limitations.

## 4. Discussion

### 4.1. Screen Exposure Trends

The first aim of this study was to describe current trends in screen exposure among preschool children. The results indicate a high prevalence of digital devices in households, with nearly all families owning mobile phones, televisions, and laptops, which is consistent with both international findings [6,38] and previous Croatian research [7]. Differences in the distribution of specific devices were observed, with a somewhat higher prevalence of laptops and tablets and a lower prevalence of personal computers compared to earlier studies [25,38]. A novel contribution of this study is the inclusion of virtual reality devices, which were present in a small proportion of households, although their use among children remains limited. Compared to research from 2020, there has been an increase in the prevalence of this device in households in general, but a decrease in the use of virtual reality headsets among children [7]. Additionally, approximately one in ten children owned a personal mobile device, in line with national data [7].

Regarding screen exposure duration, the findings suggest that children generally did not exceed recommended guidelines, particularly during weekdays, while weekend exposure was somewhat higher but still within recommended limits [36]. These results are broadly consistent with previous international and regional studies [39,40,41], although some variation in weekend exposure was observed.

### 4.2. Correlation Between Temperament, Screen Exposure, and Psychological Adjustment

To examine the relationships among temperament dimensions, screen exposure, and psychological adjustment in preschool children, correlation analyses were conducted. Externalizing problems were positively associated with Emotionality and Activity and showed a strong positive association with Impulsivity (Table 5). Internalizing problems were positively related to Emotionality and Impulsivity, but negatively related to Sociability. In addition, prosocial behavior was negatively associated with Impulsivity, indicating that less impulsive children exhibited more prosocial behaviors. Overall, these findings highlight Impulsivity as the most consistent correlate of all dimensions of psychological adjustment. These findings are largely consistent with previous results by Valiente et al. [11], who reported that effortful control was negatively correlated with the occurrence of externalizing problems in children, which can be considered the opposite dimension of Impulsivity as measured by the EASI questionnaire. Previous studies have also found that Impulsivity positively correlates with externalizing problems [42,43]. At the same time, Eisenberg and Fabes (1992, as cited in [11]) revealed the role of negative emotionality in “strengthening” the relationship between regulation and externalizing problems, in a way that children with reduced regulation and elevated levels of negative emotionality were most prone to externalizing problems. The findings are consistent with the research of Santens et al. [44], who report that low levels of effortful control predict the occurrence of internalizing problems such as anxiety and depression. It should be emphasized that Sociability did not correlate with any other variables, except for internalizing problems, which may be explained by the subscale’s low reliability in this sample. Still, these results are similar to research by Stanhope et al. [45], which shows no significant association between Sociability and helping behavior, as measured through mothers’ reports. Finally, Andrade and Tannock [46] found that symptoms of Impulsivity significantly predicted peer relationship problems, which confirms the observed negative association between Impulsivity and prosocial behavior in this sample.

Furthermore, in this study, weekday screen exposure was not related to any dimension of psychological adjustment, except for a nonlinear relationship with prosocial behavior, contrary to previous findings suggesting that longer screen time predicts behavioral difficulties [22,47,48]. This discrepancy may be explained by the relatively small and narrow sample size, the skewed distribution of behavioral problems, and reliance on parental estimates of screen time from a non-validated questionnaire, all of which could have led to observing lower and non-significant correlations.

As for weekend exposure, the item showed a significant positive correlation with externalizing problems and a significant negative correlation with children’s prosocial behavior, consistent with previous findings: increased screen exposure predicts greater externalizing and lower prosocial behavior [22,49]. One potential reason is that time spent on screens replaces real interactions with peers, so children may not have the opportunity to display prosocial behaviors [49]. In addition, some children who rely heavily on screens for entertainment and communication may become less comfortable with face-to-face interactions and increasingly prefer online contact over in-person relationships [50]. Also, limiting or removing devices can trigger intense anger and distress (“device withdrawal tantrums”) [51,52], reflecting difficulties in emotion regulation after screen removal. This may also spill over into peer relationships, for example, through increased irritability or conflict in offline interactions. Excessive screen time may also be negatively associated with developmental outcomes, particularly when it replaces time that would otherwise be spent playing. More precisely, Berk [53] emphasizes that between the ages of six and eleven, children engage in an increasing number of rule-based joint games and spend most of their time in some form of physical activity. Thus, children enjoy activities such as running, jumping, and hopping, while, over time, they incorporate increasingly developed cognitive and social skills, progressing from simple racing games to games such as “tag”, “chain tag”, and “frozen queen” [53]. The mentioned games are considered age-appropriate and essential for mastering developmental goals, but in the home environment, they can sometimes be seen as inappropriate. More precisely, it is possible to assume that children’s level of activity and their need to develop flexibility, spatial coordination, and balance may be interpreted as a type of externalizing problem if such behavior is expressed at home. Furthermore, a child who spends most of their time alone, looking at a device, does not have as well-developed physical skills as other children, which may lead to social comparisons, a lack of self-confidence, and ultimately the expression of externalizing problems.

Finally, differences in the relationships between weekend and weekday exposure durations and children’s psychological adjustment may be explained by the fact that all children in the sample attend kindergarten during the week. In contrast, on weekends, they spend most of their time with their parents. Therefore, it is possible to expect more accurate and detailed estimates from mothers when they spend more time with their children, compared to times when children spend most of their waking hours in kindergarten. Also, it can be assumed that children spend a similar amount of time playing in kindergarten, while the ratio of time spent on play and interaction with peers to time spent in front of screens on weekends is unknown. It is therefore possible that some mothers notice more externalizing problems and fewer prosocial behaviors when the child spends most of the weekend at home, in front of screens, whereas other mothers notice more prosocial behaviors and fewer externalizing problems when the child spends more time outside the home.

In accordance with all the above, it can be observed that the obtained findings on trends, mainly in weekend screen exposure, generally agree with previous findings in Croatia [7,25] and worldwide [26,54], as well as findings on the relationship between temperament, screen exposure, and psychological adjustment [11,49]. Accordingly, it is considered justified to devote attention to a more detailed investigation of the nature of this relationship. Moderation analyses were therefore included in the study to investigate the role of screen exposure in the relationship between temperament and psychological adjustment.

### 4.3. Exploring the Moderating Effect of Weekend Screen Time on the Relationship Between Temperament and Psychological Adjustment

Hierarchical regression analyses testing the moderation model predicting externalizing problems showed that Impulsivity was a significant predictor and that the interaction with weekend screen exposure was also significant. These findings suggest that weekend screen exposure may alter the relationship between Impulsivity and externalizing problems, such that the association between the two appears weaker at higher levels of weekend screen exposure. The findings thus suggest that, in more impulsive children, the duration of screen exposure may reduce the likelihood of engaging in undesirable behaviors. The reason for this may be that screen exposure provides an alternative form of behavior, reducing the likelihood that the child will engage in risky behaviors during that time. More precisely, screen exposure may act as a distraction, reducing the likelihood of engaging in physical activity in general and in new social situations where the child would otherwise exhibit externalizing problems. An additional explanation of this relationship may be that on weekends, parents are more involved in children’s viewing of screen content, so their presence and discussion of the content may lead to a reduction in the strength of this relationship by encouraging learning related to empathy, prosocial behavior, and positive peer relationships.

Similarly, weekend screen exposure moderated the association between Activity and externalizing problems, with this association being weaker at higher levels of exposure. This can again be explained by the very nature of screen exposure, which is why the World Health Organization [55] refers to it as “sedentary screen-based activities”. Namely, standard consumption of screen content involves sitting, inactivity, and possibly “typing”. Accordingly, the results suggest that the association between Activity and externalizing problems weakens with longer screen exposure durations. One possible explanation is that activity itself requires rapid task execution and generally an accelerated pace [28]. The content itself being played may affect children’s activity levels, because watching animated series and films for children encourages focused attention and active listening, thereby promoting “quiet sitting” and sustained engagement in a single activity. As stated, the presence of parents near children and joint viewing of media content may indirectly affect children’s activity, as parents are more likely to direct and calm them when they are in their immediate vicinity. An additional explanation for the weaker association between Activity and externalizing problems at higher levels of screen exposure relates to children’s initial motivation to engage with screen content. This would explain why, at low exposure levels, the connection between the mentioned constructs is stronger: it can be assumed that children with an elevated level of Activity begin watching media content but soon independently give it up and move on to the next activity. From a methodological standpoint, the subscale of externalizing problems comprises the Hyperactivity scale, which is positively associated with attention-deficit hyperactivity disorder, and this pattern is consistent with the idea that children high in Activity and hyperactivity may be less likely to remain engaged with screen content for long periods [56].

No significant moderating interaction between screen exposure duration and Emotionality in predicting externalizing problems was found. Instead, Emotionality emerged as an independent predictor: higher Emotionality scores were associated with higher levels of externalizing problems, whereas weekend screen exposure was not a significant independent predictor. Although this pattern contrasts with some previous findings on screen exposure and externalizing behavior, it may reflect the nature of the Emotionality construct, which captures children’s rapid and intense reactions and immediate responses to new or changing situations. More precisely, Buss [57] himself defines Emotionality as discomfort or distress triggered by the autonomic nervous system reactions, which, over time, takes the form of anger or fear. As such, it is unlikely to be linked to long-term screen exposure, but it may offer insight into how children react when transitioning between activities. More precisely, with a detailed understanding of the construct of Emotionality, it indicates that this dimension of temperament could be more involved in understanding the child’s behavior when beginning or ending activities, including starting or stopping device use and the intense emotional reactions (e.g., anger or “device withdrawal” tantrums) that some children show when screens are removed [51,52].

Finally, a significant moderation effect of weekend screen exposure was observed in the relationship between Impulsivity and prosocial behavior, such that longer exposure was associated with a weaker relationship between these variables. Although both impulsivity and screen exposure were independently associated with lower levels of prosocial behavior (Table 5), increased screen exposure appears to attenuate the strength of this association, suggesting that environmental factors may partially override individual differences in temperament. More specifically, it may be assumed that children with higher levels of impulsivity, when exposed to screens for longer periods, exhibit fewer prosocial behaviors. One possible explanation is that greater screen exposure may be associated with increased sensation-seeking tendencies, as suggested by findings in adult populations [58]. This may further contribute to a preference for highly stimulating environments and avoidance of situations that require regulation without immediate personal reward. Consequently, everyday social interactions may be experienced as less engaging, slower, or insufficiently stimulating, leading to reduced involvement in social exchanges and prosocial behaviors, which typically require reflection and intentional action. Furthermore, individuals with higher levels of impulsivity have been shown to exhibit patterns resembling problematic or addictive engagement with screen-based content [59], which may reduce opportunities for peer interaction and, consequently, the expression of prosocial behavior. Taken together, these findings highlight the importance of examining daily patterns of screen exposure in relation to children’s overall functioning. Disruptions in routines and reduced engagement in offline activities may negatively impact socio-emotional development. Conversely, reducing screen exposure may promote greater parent–child interaction by providing opportunities for joint activities such as play and reading, which can facilitate the learning and modeling of prosocial behaviors. These findings also highlight the importance of considering screen exposure not only as an independent predictor but also as a contextual factor that may moderate the expression of temperament in early childhood.

However, the results of this study should be interpreted with caution, given several methodological issues. Firstly, the use of an unvalidated parent-report Screen Exposure Questionnaire, which could be prone to under- or overestimating children’s screen time, could have obscured true and expected associations and produced lower effect sizes. Secondly, the low internal consistency of the Sociability scale, as well as some SDQ dimensions, could have reduced the magnitude of correlations of these constructs and made it more difficult to detect statistically significant effects. In addition, given the highly educated and homogeneous sample in this study, it is possible that the mothers were more aware of the guidelines and potential negative effects of screen use and therefore paid closer attention to their children’s screen time, resulting in lower levels of screen exposure. Also, because levels of internalizing and externalizing problems were relatively low, indicating that the families in this sample were low risk, it is questionable whether the present findings can be generalized to more socioeconomically diverse populations. This low variability may have also led to lower observed correlations, smaller effect sizes, and reduced power to detect statistically significant associations.

### 4.4. Limitations and Directions for Future Research

This study provides a basis for expanding knowledge of the relationships among a child’s stable characteristics, their social environment, and developmental outcomes. However, it is necessary to comment on certain limitations to improve future research. The greatest criticism of this study is the reduced representativeness of the sample, due to the recruitment method and the mothers’ uneven socio-demographic characteristics. Thus, over 85% of mothers with higher education were included in the sample, which deviates from the proportion of highly educated individuals in the Republic of Croatia, which amounted to 24.1% of the total population according to the 2021 census [60]. Representation also differs somewhat in the City of Zagreb, where 39.1% of the population has completed higher education, according to 2021 data from the Croatian Bureau of Statistics [60]. The sample was further reduced due to the timing of data collection, which coincided with the start of the summer holidays and children leaving kindergarten. In addition, the large response of kindergartens in the narrower center of Zagreb, which contributed to an extremely high proportion of highly educated, highly paid, and employed mothers, could lead to an incorrect conclusion about the financial situation of Zagreb residents. On the other hand, the sample also includes fewer mothers who have completed primary and secondary education, as well as some who are unemployed. According to official 2021 data, the proportion of residents with primary and secondary education is around 20.4%, while the proportion of unemployed residents is 4.6%, which differs from the sample data [60]. All of the above lead to the conclusion that the sample is narrow and that the results cannot be generalized. It is therefore suggested that more extensive research be conducted throughout Croatia so that the conclusions could be applied to the wider population. At the same time, the relatively homogeneous and highly educated sample may have reduced the influence of certain sociodemographic confounding factors, such as socioeconomic status, parental education, and variability in the family environment, allowing for a clearer examination of the relationships among the variables studied. However, this also highlights the need for future research in more socioeconomically diverse populations, where both patterns of screen exposure and developmental risks may differ.

A related concern is the reliance on maternal reports for all study variables, which introduces the risk of common method bias. Shared method variance may have inflated the observed correlations between these constructs. It is therefore recommended that future research expand the participant pool and employ multi-informant designs incorporating fathers and kindergarten teachers. Fathers may provide more accurate assessments of screen-based activities, while kindergarten teachers can assess children’s behavior during peer interactions and more precisely capture latent dimensions of temperament through direct observation. It also encourages the development of studies that include direct data collection from children, modeled after the UNICEF [40] and EU Kids Online [61] studies. It is recommended that researchers provide support to children when responding, to include older children in the first and third grades of primary school, when the effects might be even more pronounced, and to cover data beyond the scope of limitations associated with parental assessment. Furthermore, it is necessary to emphasize that the study is correlational, which precludes causal conclusions [62]. Therefore, it is suggested that further quasi-experimental research be conducted, in which content and exposure time are manipulated. Additional caution should also be exercised with the questionnaire, which relies on independent interpretation of items and subjective assessments [62]. The reason for this may lie not only in a mere misunderstanding of the question, but also in the entire process of retrieving answers from memory, aligning them with the offered possibilities, and ultimately adapting the answer due to social desirability [62]. It is also necessary to take into account the possibility that the findings go in the direction of socially desirable responses, because a tendency toward responses indicating lower exposure was observed, which can somewhat be justified by the desire of participants to present themselves in a good light and, ultimately, as “good parents”. Additional factors that may have contributed to responses ranging from “Rarely” to “Never” include participants’ response styles and a possible lack of motivation due to the questionnaire’s length. Furthermore, administering the questionnaire in person reduces the feeling of anonymity, while the place of completion itself, that is, the conduct of the research within a health institution or a kindergarten, may arouse participants’ interest in confirming their own assumptions about the aim of the research and presenting themselves in such a light [62].

Furthermore, in this study, an unvalidated Screen Exposure Questionnaire was used, which may have affected the results obtained. More precisely, given that this is the first use of the questionnaire, no pilot study was conducted, and no standardized norms were available. Also, the questionnaire’s reliability and validity were not assessed. The questionnaire primarily assesses device ownership and parental estimates of screen time duration. Although traditional psychometric indices (e.g., internal consistency, factor structure) are less applicable to such items, criterion validity is critical and was not established. Consequently, findings related to screen exposure should be interpreted with caution, as measurement error in parental estimates may have distorted the observed associations. The moderating effects of screen time, although statistically significant, require replication using validated measures before firm conclusions can be drawn. Future studies should validate parental screen-time estimates against objective measures, such as device-based tracking applications or structured time-use diaries, to establish criterion validity. Second, several scales showed low internal consistency—the EASI Sociability subscale (α = 0.54) and several SDQ subscales (α < 0.60)—which likely attenuated associations and reduced power. To address SDQ reliability issues, subscales were combined into wider dimensions. The lack of findings for Sociability may partly reflect measurement error. It is recommended to use measures with stronger psychometric properties in future studies.

Additionally, the moderation models did not control for sociodemographic variables such as maternal education, family income, household composition, or urbanization level. These variables were measured and reported descriptively (see Table 1 and Table 2) but were not included as covariates to maintain parsimony and preserve statistical power to detect the primary interaction effects of interest. Including multiple covariates in moderation analyses with samples of this size risks overfitting and can obscure the detection of interaction terms, which were central to the study’s aims. Larger samples should be included in future studies to explore whether these findings hold when controlling for family-level socioeconomic factors. It is also necessary to examine the relationship between exposure during the week and the weekend, as this can obscure the association between exposure and psychological adjustment. It is possible to hypothesize that children are equally exposed to screens during the week, while weekend exposure varies and affects preschool children’s adjustment. Finally, it is suggested that special attention be paid to language expression, as the included population does not frequently complete psychology questionnaires. An example of this is the term “immediate family,” which participants often identified as a source of ambiguity.

## 5. Conclusions

This study provides insight into current trends in screen exposure among Zagreb preschool children, revealing that almost every household contains a television and a mobile phone, followed by laptops, tablets, and personal computers. However, preschool children most frequently use mobile phones or watch television, while they rarely use other devices. Ultimately, preschool children are exposed to screens for more than one hour every day of the week. These findings generally confirm previous research and align with the correlational analyses. More precisely, it was confirmed that externalizing problems are weakly to moderately positively correlated with Emotionality and Activity, and highly positively correlated with Impulsivity. This suggests that preschool children with higher levels of Emotionality, Activity, and Impulsivity also display more externalizing problems. Furthermore, externalizing problems are weakly positively correlated with the duration of weekend screen exposure, indicating that longer exposure is associated with a higher number of observed externalizing problems. Internalizing problems are weakly and positively correlated with Emotionality and Impulsivity, and weakly and negatively correlated with Sociability, indicating that more emotionally and impulsively reactive children, as well as children with lower sociability, display more internalizing problems. Finally, prosocial behavior is weakly and negatively correlated with the Impulsivity dimension and with all measures of exposure duration. Moderation analyses of these relationships revealed that longer weekend exposure weakens the relationships between externalizing problems and Impulsivity, and between externalizing problems and Activity, whereas no significant moderating effect of weekend exposure duration was found on the relationship between Emotionality and externalizing problems. Lastly, longer exposure weakens the relationship between Impulsivity and prosocial behavior, once again highlighting the importance of further research in this area.

Finally, the present study largely confirms existing knowledge and extends scientific understanding through a unique design that examines the relationship between stable characteristics (temperament) and situational factors (screen exposure) in relation to children’s psychological adjustment. Evidence of the moderating role of time-based screen exposure in the relationship between temperament dimensions and psychological adjustment deepens knowledge in this field, opens the way for new research, and provides an opportunity to understand this specific relationship further.

## Figures and Tables

**Table 1:** Educational level of mothers (N = 105).

Education Level	f	%
Postgraduate education	21	19.8
Graduate (Master’s level) education	53	50
Undergraduate/professional higher education	19	17.9
Secondary education	11	10.4
Primary education	1	0.9

Note: f = frequency.

**Table 2:** Self-assessed family income level relative to the national average in Croatia (N = 106).

Income Level	f	%
Far above average	6	5.7
Above average	53	50
Average	40	37.7
Below average	6	5.7
Far below average	1	0.9

Note: f = frequency.

**Table 3:** Descriptive statistics of device use frequency (N = 106).

Device	C	q	min	max
Television	4	2	1	5
Mobile phone	2	2	1	5
Tablet	1	2	1	5
Laptop	1	0	1	3
Personal computer	1	0	1	4
Gaming console	1	0	1	5
Virtual reality headset	1	0	1	3

Note: C = median; q = interquartile range (IQR); min = minimum observed value; max = maximum observed value.

**Table 4:** Descriptive statistics of temperament dimensions, screen time, and psychological adjustment.

Variable	N	M	SD	min	max	S-W(p)	S	K
Internalizing problems	106	1.64	2.02	0	10	0.78 **	1.81	3.59
Externalizing problems	106	4.10	2.90	0	13	0.93 **	0.87	0.36
Prosocial behavior	105	8.56	1.62	2	10	0.82 **	−1.19	1.42
Emotionality	103	12.07	3.26	5	21	0.98	0.30	−0.18
Activity	103	15.56	3.45	7	24	0.98	0.37	−0.10
Sociability	106	18.60	2.56	8	24	0.95 **	−0.85	1.97
Impulsivity	104	12.77	3.08	7	21	0.98	0.21	−0.37
Screen time (weekday)	106	5.34	3.91	0	23	0.90 **	1.32	2.96
Screen time (weekend)	106	2.64	1.84	0	9	0.91 **	1.14	1.50

Note: N = number of participants included; M = mean; SD = standard deviation; min = minimum observed value; max = maximum observed value; S-W = Shapiro–Wilk test of normality; S = skewness; K = kurtosis; ** *p* < 0.01.

**Table 5:** Correlations between temperament dimensions, screen exposure, and psychological adjustment in preschoolers (N = 103–106).

Variables	I.B.	E.B.	P.B.	E	A	S	I	WD	WE
I.B.	1								
E.B.	0.33 **	1							
P.B.	−0.25 *	−0.29**	1						
E	0.34 **	0.35 **	−0.10	1					
A	0.03	0.47 **	−0.01	0.25 *	1				
S	−0.32 **	0.11	0.13	−0.13	0.24 *	1			
I	0.39 **	0.67 **	−0.23 *	0.48 **	0.47 **	−0.03	1		
WD	0.12	0.05	−0.21 *	0.06	−0.20 *	0.08	0.20 *	1	
WE	0.18	0.22 *	−0.22 *	0.18	−0.06	−0.02	0.34 **	0.55 *	1

Note: I.B.—internalizing behavior problems; E.B.—externalizing behavior problems; P.B.—prosocial behavior; E—Emotionality; A—Activity; S—Sociability; I—Impulsivity; WD—weekday screen exposure; WE—weekend screen exposure; *p* < 0.05 *; *p* < 0.01 **.

**Table 6:** Moderation effect of weekend screen time on the relationship between Impulsivity and externalizing problems (N = 104).

Predictor	b	SE	β	t	CI	Tolerance	VIF
Impulsivity	0.63	0.07	0.67	8.63 **	[0.49, 0.78]	0.88	1.13
Screen time (weekend)	0.04	0.13	0.03	0.34	[−0.21, 0.29]	0.83	1.21
Impulsivity × Screen time (weekend)	−0.08	0.04	−0.16	−2.19 *	[−0.16, −0.01]	0.94	1.07
R = 0.69							
R^2^ = 0.474 ΔR^2^ = 0.025 *							
F (3, 100) = 29.98 ** ΔF (1, 100) = 4.78 * Cohen’s f^2^ = 0.05 (small effect)							

Note: b = unstandardized coefficient; SE = standard error; β = standardized coefficient; t = t-value; *p* = significance level, CI = confidence interval; VIF = variance inflation factor. * *p* < 0.05, ** *p* < 0.01.

**Table 7:** Moderation effect of weekend screen time on the relationship between Activity and externalizing problems (N = 103).

Predictor	b	SE	β	t	95% CI	Tolerance	VIF
Activity	0.38	0.07	0.46	5.59 **	[0.24, 0.5]	0.99	1.02
Screen time (weekend)	0.36	0.13	0.23	2.8 **	[0.10, 0.61]	0.99	1.01
Activity × Screen time (weekend)	−0.1	0.03	−0.28	−3.39 **	[−0.16, −0.04]	0.99	1.02
R = 0.596							
R^2^ = 0.355 ΔR^2^ = 0.075 **							
F (3, 99) = 18.196 ** ΔF (1, 99) = 11.47 ** Cohen’s f^2^ = 0.12 (small-to-medium effect)							

Note: b = unstandardized coefficient; SE = standard error; β = standardized coefficient; t = t-value; *p* = significance level; CI = confidence interval; VIF = variance inflation factor; ** *p* < 0.01.

**Table 8:** Moderation effect of weekend screen time on the relationship between Impulsivity and prosocial behavior (N = 103).

Predictor	b	SE	β	t	95% CI	Tolerance	VIF
Impulsivity	−0.1	0.05	−0.19	−1.93	[−0.20, 0.003]	0.88	1.14
Screen time (weekend)	−0.07	0.09	−0.07	−0.73	[−0.24, 0.11]	0.82	1.21
Impulsivity × Screen time (weekend)	−0.08	0.03	−0.3	−3.13 **	[−0.13, −0.03]	0.94	1.07
R = 0.398							
R^2^ = 0.158 ΔR^2^ = 0.083 **							
F (3, 99) = 6.20 ** ΔF (1, 99) = 9.82 ** Cohen’s f^2^ = 0.10 (small-to-medium effect)							

Note: b = unstandardized coefficient; SE = standard error; β = standardized coefficient; t = t-value; *p* = significance level; CI = confidence interval; VIF = variance inflation factor; ** *p* < 0.01.

## Data Availability

The data presented in this study are available on request from the corresponding author. The data are not publicly available due to privacy and ethical reasons.
